# Joint Effects of Anticholinergic Burden and Neurofilament Light on Dementia Risk: The Shanghai Aging Study

**DOI:** 10.1002/cns.70691

**Published:** 2025-12-10

**Authors:** Danyi Chi, Xiaoniu Liang, Zhenxu Xiao, Xiaowen Zhou, Qianhua Zhao, Bin Wang, Ding Ding

**Affiliations:** ^1^ Department of Pharmacy Huashan Hospital, Fudan University Shanghai China; ^2^ Institute of Neurology Huashan Hospital, Fudan University Shanghai China; ^3^ National Clinical Research Center for Aging and Medicine Huashan Hospital, Fudan University Shanghai China; ^4^ National Center for Neurological Disorders Huashan Hospital, Fudan University Shanghai China

**Keywords:** anticholinergic drugs, cohort study, dementia, neurodegeneration burden, neurofilament light chain, older adults

## Abstract

**Aims:**

Anticholinergic drugs (ACDs) and the neurodegeneration biomarker neurofilament light chain (NfL) are associated with dementia; however, the interplay between anticholinergic drug exposure and neurodegeneration in dementia risk remains underexplored.

**Methods:**

This prospective cohort study analyzed 1529 dementia‐free adults (median follow‐up 5.2 years) from the Shanghai Aging Study. Cumulative anticholinergic burden was quantified using the anticholinergic cognitive burden (ACB) scale and total standardized daily dose (TSDD) over 1 year pre‐baseline. Neurofilament light chain (NfL) levels were assayed via single‐molecule array (Simoa).

**Results:**

Elevated NfL (adjusted HR 1.77, 95% CI, 1.07–2.92) and TSDD exposure (HR 1.55, 1.08–2.24) were independently associated with incident dementia risk. Participants with both TSDD exposure and high NfL levels showed substantially greater cumulative dementia incidence versus those with no TSDD/low NfL (log‐rank *p* < 0.0001; adjusted HR 2.24, 1.20–4.20). Individuals with both high TSDD and high NfL demonstrated a significantly higher dementia risk (HR 6.34, 95% CI, 1.90–21.20) compared to low‐burden counterparts.

**Conclusions:**

These findings identify plasma NfL as a critical modifier of anticholinergic‐related cognitive vulnerability, providing mechanistic insights for risk stratification and supporting biomarker‐guided deprescribing strategies in older adults exposed to ACDs.

## Graphical abstract

The combined ACD use and elevated NfL levels jointly increased the risk of incident dementia. Older adults with both high ACDs exposure and high NfL levels exhibited a significantly higher risk of dementia compared to low‐burden counterparts.

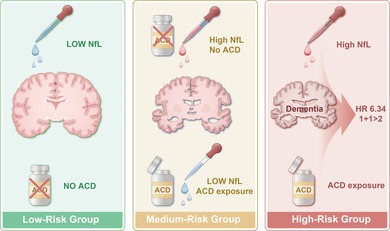

## 1 Introduction

Anticholinergic drugs (ACDs) are widely prescribed for conditions such as mental, neurological, respiratory, ophthalmic, and urological disorders, with prevalence rates ranging from 11% to 80% in older adults [1]. ACDs block the neurotransmitter acetylcholine in the central or peripheral nervous system, with effects varying by site. Dementia affects over 50 million globally, projected to triple to 153 million by 2050, with modifiable risk factors explaining only 40% of cases, highlighting critical prevention research gaps [2]. While the acute cognitive impairment effects of ACDs are well documented, their long‐term impact on dementia remains controversial. Some studies report a dose‐dependent relationship between ACD use and dementia risk [3, 4, 5, 6], whereas others find no significant association [7, 8].

Neurofilament light chain (NfL), a subunit of neurofilaments, is a biomarker for axonal injury and neurodegeneration [9]. Elevated blood NfL levels are associated with cognitive decline and dementia, making it a promising diagnostic and prognostic tool [10].

Beyond the independent health effects of ACDs and NfL, the potential joint effects of them on dementia are underexplored. The Shanghai Aging Study, a prospective cohort study, provides an opportunity to investigate this relationship [11, 12]. Our previous study found a dose–response relationship of possible ACD use with the risk of dementia only in APOE ε4 carriers [4]. In this study, we analyzed the 5 year longitudinal data to verify the hypothesis that there was a joint association of ACD use and elevated NfL levels with incident dementia.

## 2 Materials and Methods

### 2.1 Study Population

This prospective cohort analysis utilized data from the Shanghai Aging Study, which recruited community‐dwelling adults aged ≥ 60 years in Jingansi community, Shanghai (2010–2011). Detailed study procedures were published elsewhere [11, 12]. Inclusion criteria of the current study were: (1) dementia‐free at baseline with completed cognitive assessments; (2) follow‐up until dementia diagnosis or March 2017; (3) available baseline blood samples.

The study protocol was approved by the Medical Ethics Committee of Huashan Hospital, Fudan University (Approval Number: 2009‐195). Written informed consent was obtained from all individuals or legal guardians.

### 2.2 Anticholinergic Drug Exposures Assessment

Data on medication use (prescription, over‐the‐counter, and supplements) within the year preceding baseline were collected via structured interviews and cross‐verified with medical records to capture a period of recent and likely continuous use, thus minimizing recall bias from more distant historical use. ACDs were classified using the Anticholinergic Cognitive Burden (ACB) scale: ACB = 1 (possible ACDs, in vitro anticholinergic activity) and ACB ≥ 2 (definite ACDs, clinically relevant peripheral/central effects). Cumulative anticholinergic burden was quantified as the total standardized daily dose (TSDD), calculated as:

$$\text{TSDD}=\sum \left(\;\frac{\text{Units dispensed}\times \text{Strength}\;\left(\mathrm{mg}\right)}{\text{Minimum Effective Geriatric Dose}}\times \mathrm{ACB}\;\text{weight}\right)$$

The minimum effective geriatric dose (MEGD), as defined by a geriatric medication reference [13] and this TSDD calculation method aligns with prior validation studies [4, 6].

### 2.3 Laboratory Tests

Plasma neurofilament light chain (NfL) levels at baseline were measured using single‐molecule array (Simoa) technology on the HD‐X platform (Quanterix), following manufacturer protocols. Operators were blinded to clinical data. APOE genotyping was performed via TaqMan SNP assay (Thermo Fisher Scientific), with ε4 carriers defined as having ≥ 1 ε4 allele [14].

### 2.4 Neuropsychological Assessments and Dementia Diagnosis

Cognitive function was evaluated using a standardized battery, covering domains of global cognition, executive function, spatial construction, memory, language, and attention [12]. Participants with < 6 years of education received simplified versions of selected tests, while those with ≥ 6 years completed additional tasks. The intra‐rater and inter‐rater reliability for the neuropsychological assessments has been reported in our previous study [12].

Mood assessments incorporated the Zung Self‐Rating Anxiety Scale (ZSAS) [15] and the Center for Epidemiologic Studies Depression Scale (CESD) [16]. Anxiety was defined as ZSAS > 44 and depression as CESD ≥ 16, thresholds established in prior psychometric studies [12]. Functional status was measured via the Lawton‐Brody Activity of Daily Living (ADL) scale [17]. Participants were classified as functionally intact if ADL scores > 16.

Neurological examinations were conducted by certified neurologists. A consensus panel reviewed medical histories, neuropsychological results, functional assessments, and psychiatric evaluations to diagnose dementia according to DSM‐IV criteria [18]. Participants were followed from April 2014 to March 2017, with cognitive function re‐evaluated using the same neuropsychological battery and the same diagnostic criteria.

### 2.5 Statistical Analysis

Continuous variables were summarized as mean ± standard deviation (SD), categorical variables as frequencies (%). The correlation between TSDD and NfL levels was evaluated using a generalized linear model (GLM). Multivariable Cox proportional hazards models estimated hazard ratios (HRs) and 95% confidence intervals (CIs) for dementia risk based on TSDD and NfL, adjusted for age, sex, and APOE genotype. The proportional hazards assumption was validated by plotting Schoenfeld residuals. Cumulative dementia incidence was visualized using Kaplan–Meier curves, with between‐group differences assessed via log‐rank tests.

Total standardized daily dose (TSDD) exposure was categorized as TSDD = 0 (no exposure) versus TSDD > 0 (exposure), and NfL levels were dichotomized into high and low groups based on the median value (15.68 pg/mL). To examine joint effects, participants were stratified into three mutually exclusive risk groups: (1) high‐risk group: TSDD > 0 and high NfL; (2) medium‐risk group: TSDD = 0 and high NfL, or TSDD > 0 and low NfL; and (3) low‐risk group: TSDD = 0 and low NfL.

Among ACD users, TSDD was further dichotomized into low and high levels based on the median (365), and the joint risk groups were similarly defined.

All analyses were performed using SAS 9.4 (SAS Institute), with significance set at *p* < 0.05.

## 3 Results

### 3.1 Baseline Characteristics of Participants

**FIGURE 1:**
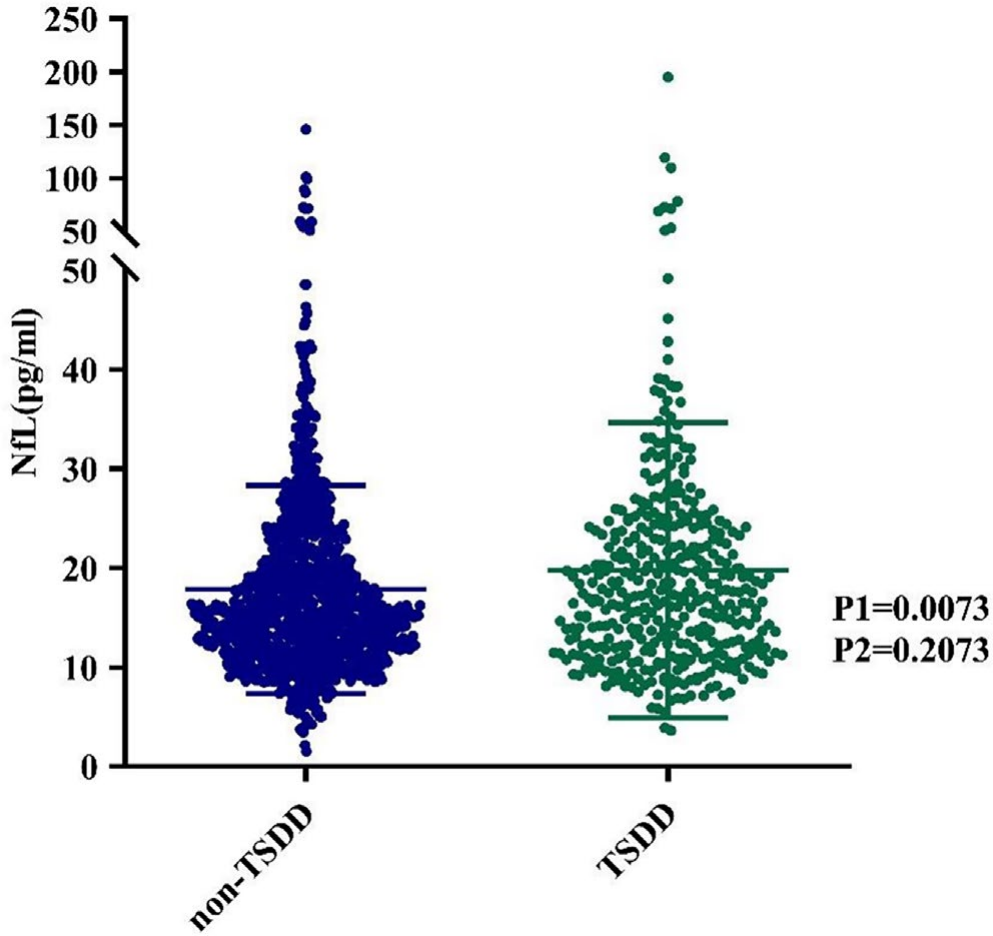
The distribution of blood NfL concentrations between participants with no TSDD and participants with TSDD. (P1) Univariate analysis of the NfL concentrations between participants with no TSDD and participants with TSDD. (P2) Comparisons of the blood NfL concentrations between participants with no TSDD and participants with TSDD adjusted by age, sex, and APOE. APOE, ε4+, apolipoprotein E ε4 positivity; NfL, neurofilament light chain; TSDD, total standardized daily dose.

As shown in Figure 1 and Table 1, among 1529 participants without dementia at baseline, 124 developed dementia during the median follow‐up of 5.2 years (interquartile range: 4.8–5.8 years). Of these, 1136 participants had no TSDD of ACDs, while 393 (25.7%) participants had TSDD exposure (202 with low TSDD and 191 with high TSDD). Compared with participants without TSDD exposure, those with TSDD were older and had higher NfL concentrations at baseline (*p* < 0.0001). Similarly, participants with high TSDD were older than those with low TSDD (*p* = 0.023).

**TABLE 1:** Baseline characteristics of participants.

	Total (*n* = 1529)	Non‐TSDD (*n* = 1136)	TSDD (*n* = 393)	Low TSDD (*n* = 202)	High TSDD (*n* = 191)	*p* ^a^	*p* ^b^
Age, year	70.72 ± 6.87	70.21 ± 6.82	72.20 ± 6.82	71.41 ± 7.02	73.03 ± 6.53	< 0.0001	0.0230
Sex, female (%)	815 (53.30)	625 (55.02)	190 (48.35)	105 (51.98)	98 (51.31)	0.0223	0.8941
Education, year	12.00 ± 3.95	12.01 ± 3.87	11.97 ± 4.20	12.11 ± 4.13	11.82 ± 4.29	0.6737	0.5640
MMSE, score	28.32 ± 1.93	28.38 ± 1.82	28.13 ± 2.19	28.27 ± 1.89	27.98 ± 2.46	0.0526	0.6494
NfL, pg/mL	18.33 ± 11.80	17.83 ± 10.49	19.79 ± 14.89	19.71 ± 16.97	19.87 ± 12.37	0.0073	0.3939
TSDD	153.72 ± 360.92	0.00 ± 0.00	598.06 ± 491.27	290.82 ± 95.19	923.00 ± 530.89	< 0.0001	< 0.0001
APOE, ε4 + (%)	249 (16.76)	191 (17.35)	58 (15.06)	33 (16.67)	25 (13.37)	0.3019	0.3660

*Note:* The continuous data are presented as mean ± standard deviation (SD), and the categorical data are presented as *n* (%). *p*‐value: low TSDD versus high TSDD.

Abbreviations: APOE, ε4+, apolipoprotein E ε4 positivity; MMSE, mini‐mental state examination; NfL, neurofilament light chain; TSDD, total standardized daily dose.

^a^ Comparison between groups with non‐TSDD and TSDD.

^b^ Comparison between groups with low TSDD and high TSDD.

A total of 28 ACDs were used by participants at baseline. The most commonly used possible ACDs included nifedipine (*n* = 142), hydralazine (*n* = 131), metoprolol (*n* = 122), isosorbide (*n* = 76), and captopril (*n* = 33). The most frequently used definite ACDs were promethazine (*n* = 115) (Table 2).

**TABLE 2:** Anticholinergic drugs exposure at baseline.

Possible ACDs	Number of users (%)	Definite ACDs	Number of users (%)
Nifedipine	142 (9.28)	Promethazine	115 (7.52)
Hydralazine	131 (8.56)	Chlorpheniramine	9 (0.61)
Metoprolol	122 (7.95)	Doxepin	3 (0.18)
Isosorbide	76 (4.98)	Paroxetine	3 (0.18)
Captopril	33 (2.18)	Clozapine	2 (0.12)
Triamterene	16 (1.03)	Belladonna	2 (0.12)
Digoxin	12 (0.79)	Amantadine	1 (0.06)
Theophylline	11 (0.73)	Olanzapine	1 (0.06)
Dipyridamole	8 (0.55)	Trihexyphenidyl	1 (0.06)
Prednisone	7 (0.49)	Chlorpromazine	1 (0.06)
Diazepam	6 (0.36)		
Ranitidine	6 (0.36)		
Warfarin	6 (0.36)		
Cetirizine	5 (0.30)		
Alprazolam	4 (0.24)		
Trazodone	2 (0.12)		
Cimetidine	1 (0.06)		
Furosemide	1 (0.06)		

*Note:* The percentage was calculated as the number of participants who used the drug divided by the number of all participants.

Abbreviation: ACDs, anticholinergic drugs.

### 3.2 Risk Estimations of Dementia by Baseline TSDD and NfL

As shown in Table 3, participants with high baseline NfL concentrations had a significantly higher risk of dementia compared to those with low NfL levels (HR 1.77, 95% CI, 1.07–2.92, *p* = 0.0263), after adjusting for age, sex, and APOE genotype. Similarly, participants with TSDD exposure had a higher risk of dementia compared to those without TSDD exposure (HR 1.55, 95% CI, 1.08–2.24, *p* = 0.0189) in a univariate model.

**TABLE 3:** Risk estimations of dementia by baseline TSDD and NfL.

	Model 1 HR (95% CI)	*p*	Model 2 HR (95% CI)	*p*
NfL				
Low‐NfL	1		1	
High‐NfL	4.42 (2.85, 6.87)	< 0.0001*	1.77 (1.07, 2.92)	0.0263*
TSDD				
Non‐TSDD	1		1	
TSDD	1.5 (1.08, 2.24)	0.0189*	1.36 (0.93, 1.99)	0.1107

*Note:* Model 1: univariate COX regression model; Model 2: multivariate COX regression model adjusting for age, sex, and APOE. SIgnificance of * indicates statistical significance (*p* < 0.05).

Abbreviations: 95% CI, 95% confidence interval; APOE, ε4+, apolipoprotein E ε4 positivity; HR, hazard ratio; NfL, neurofilament light chain; TSDD, total standardized daily dose.

### 3.3 Joint Effect of TSDD and NfL

**FIGURE 2:**
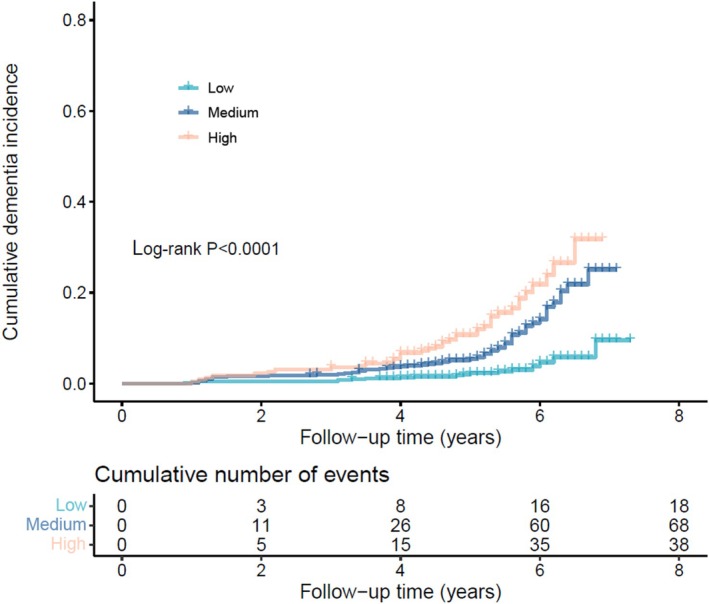
Accumulative dementia incidence in participants among low, medium, and high‐risk groups. Plasma NfL was binarized by the median. TSDD was binarized by zero and non‐zero. Low: low‐risk group, non‐TSDD and low NfL; Medium: medium‐risk group, non‐TSDD and high NfL or TSDD and low NfL; High: high‐risk group, TSDD and high NfL. NfL, neurofilament light chain; TSDD, total standardized daily dose.

For all participants, the high‐risk group (TSDD > 0 and high NfL) exhibited a significantly increased cumulative risk of dementia compared to the low‐risk group (TSDD = 0 and low NfL) (log‐rank test, *p* < 0.0001; Figure 2). After adjusting for age, sex, and APOE genotype, the high‐risk group had a higher risk of dementia (HR 2.24, 95% CI, 1.20–4.20, *p* = 0.0116) compared to the low‐risk group (Table 4).

**TABLE 4:** Joint effects of TSDD and NfL on dementia.

TSDD and NfL	Model 1 HR (95% CI)	*p*	Model 2 HR(95% CI)	*p*
Total participants				
Low‐risk group	1		1	
Medium‐risk group	3.41 (2.03, 5.74)	< 0.0001*	1.58 (0.88, 2.83)	0.1228
High‐risk group	5.80 (3.31, 10.17)	< 0.0001*	2.24 (1.20, 4.20)	0.0116*
Participants with TSDD				
Low‐risk group	1		1	
Medium‐risk group	3.32 (0.99, 11.20)	0.0527	1.09 (0.83, 1.44)	0.5301
High‐risk group	6.34 (1.90, 21.20)	0.0027*	1.09 (0.78, 1.52)	0.6137

*Note:* (1) Among total participants: Low‐risk group: Non‐TSDD and low NfL; Medium‐risk group: non‐TSDD and high NfL or TSDD and low NfL; High‐risk group: TSDD and high NfL. (2) Among participants with TSDD: Low‐risk group: Low TSDD and low NfL; Medium‐risk group: Low TSDD and high NfL or high TSDD and low NfL; High‐risk group: high TSDD and high NfL. Model 1: Univariate COX regression model; Model 2: Adjusting for age, sex, and APOE. Significance of * indicates statistical significance (*p* < 0.05).

Abbreviations: 95% CI, 95% confidence interval; APOE, ε4+, apolipoprotein E ε4 positivity; HR, hazard ratio; NfL, neurofilament light chain; TSDD, total standardized daily dose.

**FIGURE 3:**
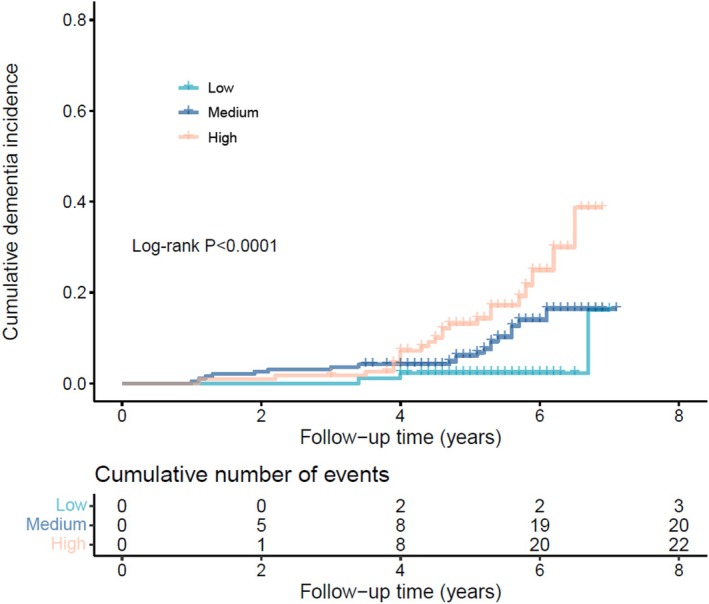
Accumulative dementia incidence in participants with TSDD among the low, medium, and high‐risk groups. Plasma NfL and TSDD were both binarized by the median. Low: low‐risk group, low TSDD and low NfL; Medium: medium‐risk group, low TSDD and high NfL or high TSDD and low NfL; High: high‐risk group, high TSDD and high NfL. NfL, neurofilament light chain; TSDD, total standardized daily dose.

Among participants with TSDD exposure, the high‐risk group (high TSDD and high NfL) showed a significantly increased cumulative risk of dementia (log‐rank test, *p* < 0.0001; Figure 3). As shown in Table 4, the univariate model indicated that the high‐risk group had a markedly higher risk of dementia compared to the low‐risk group (HR 6.34, 95% CI, 1.90–21.20, *p* = 0.0027), while the medium‐risk group did not show a significant difference. No statistical significance was found in the multivariate model.

## 4 Discussion

In this study, we demonstrated that the combined ACD use and elevated NfL levels jointly increased the risk of incident dementia in a community‐dwelling population. Older adults with both high TSDD of ACDs and high NfL levels exhibited a significantly higher risk of dementia, highlighting the importance of considering both pharmacological and biomarker profiles in dementia risk assessment. To our knowledge, this is the first population‐based study to investigate the joint effects of anticholinergic burden and axonal injury biomarkers on dementia risk, providing novel insights into the pharmaco‐biomarker interplay in neurodegeneration. It is true that due to the limited sample size, this finding remains preliminary and must be validated through future studies with sufficient statistical power.

The association between ACDs and dementia has been inconsistent across studies, likely due to variations in sample sizes, population characteristics, follow‐up durations, diagnostic criteria, and definitions of ACDs exposure [7, 19]. Some studies have reported a dose‐dependent relationship between ACD use and dementia risk, particularly for definite ACDs (HR 1.20, 95% CI, 1.15–1.26) [19]. However, others have found that ACD use primarily leads to cognitive decline without significantly increasing dementia risk [7]. Notably, large‐scale studies in the UK have shown that specific categories of ACDs, such as bladder antimuscarinic drugs (adjusted OR [AOR] 1.65, 95% CI, 1.56–1.75), antiparkinson drugs (AOR 1.52, 95% CI, 1.16–2.00), and antipsychotics (AOR 1.70, 95% CI, 1.53–1.90), are more strongly associated with dementia, while gastrointestinal drugs show no significant link [3, 5]. Recent research has increasingly focused on the role of possible ACDs in dementia. For example, Cai et al. found that older adults using at least three possible ACDs for over 90 days had a higher risk of mild cognitive impairment (OR 2.73, 95% CI, 1.27–5.87) [7]. Our previous study also identified a dose–response relationship between possible ACD use and dementia risk, particularly in APOE ε4 carriers (HR 5.71, 95% CI, 2.04–15.94) [4].

Blood NfL has emerged as a promising biomarker for neurodegeneration, with studies consistently linking elevated NfL levels to cognitive decline and dementia [20]. For instance, a prospective cohort study found that baseline NfL levels predicted AD progression over a 6 year follow‐up period [21]. Another study demonstrated that baseline NfL values could predict AD onset within an average of 22 months [22]. These findings underscore the potential of NfL as a reliable biomarker for dementia prediction [23]. Our results further support this notion, showing that participants with both high TSDD and high NfL levels had a significantly higher risk of dementia compared to those with low TSDD and low NfL. This suggests that combining pharmacological exposure (ACDs) and biomarker profiles (NfL) may improve the identification of older adults at high risk of dementia. Future priorities include achieving international standardization of assays, along with the establishment of universally accepted, context‐specific reference ranges and cut‐offs, as well as demonstrating cost‐effectiveness in large‐scale screening programs.

Evidence from basic research supports the association between ACDs and dementia. Human studies suggest that anticholinergics may exacerbate AD pathology, including amyloid plaques and neurofibrillary tangles [24], as well as increase brain atrophy and dysfunction [25, 26]. Animal models propose additional mechanisms, such as synaptic membrane alterations, reduced synaptic numbers, and inflammation, which may contribute to dementia risk independently of traditional neuropathological findings [27, 28]. Conversely, NfL, a subunit of neurofilaments, is a biomarker for neuroaxonal structural integrity. Elevated blood NfL levels are associated with hippocampal atrophy, reduced cerebral metabolic rates of glucose (CMRgl), and increased amyloid‐beta (Aβ) and tau deposition, as measured by positron emission tomography (PET) [20, 29, 30]. Based on our findings and this existing evidence, we propose a “dual‐hit” model to explain the observed synergy. In this framework, ACDs primarily induce a functional deficit by impairing cholinergic neurotransmission, which is critical for cognitive processes. Elevated NfL, in contrast, reflects an underlying structural burden of neuroaxonal injury and neurodegeneration. We hypothesize that in individuals with pre‐existing structural vulnerability (high NfL), the brain's compensatory capacity is diminished. The introduction of a cholinergic functional insult by ACDs may therefore overwhelm this compromised neural system, leading to a synergistic increase in dementia risk. This model positions ACDs and NfL as operating through complementary pathways that converge to accelerate cognitive failure, rather than positing a direct causal link between them. Genetic factors such as the APOE ε4 allele, a known modulator of Alzheimer's pathology and NfL levels, may further exacerbate this interplay. It is important to note that this mechanistic framework, while plausible and supported by indirect evidence, remains a hypothesis generated from an observational study and requires direct validation in future research. Our findings have important clinical implications. Monitoring blood NfL levels and ACD use could help identify individuals at high risk of dementia, enabling targeted interventions. For example, promethazine, the most commonly used definite ACD in our study, could be replaced with a non‐anticholinergic second‐generation antihistamine like loratadine. Similarly, hydralazine and captopril, which also carry anticholinergic risks, could be substituted with safer alternatives like losartan. For patients who cannot discontinue ACDs, regular monitoring of NfL levels and comprehensive dementia risk assessments (e.g., annual check‐ups) may help guide interventions before significant cognitive decline is evident.

Our study has several limitations. First, the diagnosis of all‐cause dementia was based on DSM‐IV criteria and neuropsychological assessments, without neuroimaging or pathologic biomarker confirmation of specific dementia subtypes. This was due to the unavailability and unacceptability of magnetic resonance imaging (MRI), cerebrospinal fluid (CSF) analysis, or PET scans for all participants. Second, the relatively small sample size and number of dementia cases limited our ability to include additional confounding variables, such as hypertension, diabetes, and depression. The potential for residual confounding from these and other unmeasured factors should be considered when interpreting our results. Third, the low proportion of definite ACD use (19.69%) and the low doses of promethazine (mean dose: 5.8 mg, compared to the MEGD of 25 mg) may have impacted the observed association between ACDs and dementia. Fourth, the lack of quantitative medication adherence data may have resulted in exposure misclassification. This most likely led to an underestimation of the true associations, meaning our risk estimates are likely conservative. Future studies should employ direct adherence monitoring to confirm these findings.

## 5 Conclusions

This is the first study to demonstrate a synergistic effect between ACD burden and elevated NfL levels on dementia risk, highlighting the importance of integrating pharmacological and biomarker data in dementia risk assessment. Our findings suggest that older adults with elevated NfL levels are particularly vulnerable to ACD‐related cognitive impairment, and plasma NfL testing may guide deprescribing of high‐risk ACDs in older adults, prioritizing non‐anticholinergic alternatives to mitigate dementia risk. Future studies with larger, more diverse populations and detailed characterization of ACD use are needed to validate our findings.

## Author Contributions

D.C. and X.L. wrote the manuscript; D.C., X.L., D.D., B.W. designed the research; Z.X., X.Z., Q.Z. performed the research; D.C., X.L., D.D., B.W. analyzed the data.

## Funding

This study was supported by the National Natural Science Foundation of China (82173599, 82473701, 82071200, 82371429), Shanghai Hospital Development Center (SHDC2020CR4007), MOE Frontiers Center for Brain Science (JIH2642001/028), STI2030‐Major Projects (2021ZD0200800), Beijing Life Oasis Public Service Center (CPHCF‐ZLKY‐2023039), and Shenzhen Ye Chenghai Charity Foundation (HIM‐202420016).

## Ethics Statement

The study protocol was approved by the Medical Ethics Committee of Huashan Hospital, Fudan University (Approval Number: 2009‐195).

## Consent

Written informed consent was obtained from all individuals or legal guardians.

## Conflicts of Interest

The authors declare no conflicts of interest.

## Data Availability

The data that support the findings of this study are available on request from the corresponding author. The data are not publicly available due to privacy or ethical restrictions.
